# Supplementary material for: Translaminar recurrence from layer 5 suppresses superficial cortical layers
Source: Nat Commun. 2022 May 11;13:2585. doi: 10.1038/s41467-022-30349-w (PMC9095870; doi:10.1038/s41467-022-30349-w)
Supplement: Supplementary file 3 — Description of Additional Supplementary Files [file 41467_2022_30349_MOESM3_ESM.pdf]

File name: Supplementary Data 1

Description: Summary of statistics used in all figures.

Number of replications, statistical test, and p value are listed for each experiment in all figures and supplementary figures.
