# Supplementary material for: Translaminar recurrence from layer 5 suppresses superficial cortical layers
Source: Nat Commun. 2022 May 11;13:2585. doi: 10.1038/s41467-022-30349-w (PMC9095870; doi:10.1038/s41467-022-30349-w)
Supplement: Supplementary file 5 — Reporting Summary [file 41467_2022_30349_MOESM5_ESM.pdf]

nature portfolio

Corresponding author(s): Hiroyuki Kato

Last updated by author(s): Mar 24, 2022

Reporting Summary

Nature Portfolio wishes to improve the reproducibility of the work that we publish. This form provides structure for consistency and transparency in reporting. For further information on Nature Portfolio policies, see our Editorial Policies and the Editorial Policy Checklist.

Statistics

For all statistical analyses, confirm that the following items are present in the figure legend, table legend, main text, or Methods section.

n/a	Confirmed	
☐	☒	The exact sample size (*n*) for each experimental group/condition, given as a discrete number and unit of measurement
☐	☒	A statement on whether measurements were taken from distinct samples or whether the same sample was measured repeatedly
☐	☒	The statistical test(s) used AND whether they are one- or two-sided<br>*Only common tests should be described solely by name; describe more complex techniques in the Methods section.*
☐	☒	A description of all covariates tested
☐	☒	A description of any assumptions or corrections, such as tests of normality and adjustment for multiple comparisons
☐	☒	A full description of the statistical parameters including central tendency (e.g. means) or other basic estimates (e.g. regression coefficient) AND variation (e.g. standard deviation) or associated estimates of uncertainty (e.g. confidence intervals)
☐	☒	For null hypothesis testing, the test statistic (e.g. *F*, *t*, *r*) with confidence intervals, effect sizes, degrees of freedom and *P* value noted<br>*Give P values as exact values whenever suitable.*
☒	☐	For Bayesian analysis, information on the choice of priors and Markov chain Monte Carlo settings
☒	☐	For hierarchical and complex designs, identification of the appropriate level for tests and full reporting of outcomes
☐	☒	Estimates of effect sizes (e.g. Cohen's *d*, Pearson's *r*), indicating how they were calculated

Our web collection on statistics for biologists contains articles on many of the points above.

Software and code

Policy information about availability of computer code

Data collection	Matlab (2013a,2016b, 2018a)<br>Bpod 1.58<br>Open Ephys 4.5<br>Bonsai 2.3.1
Data analysis	Matlab (2016b, 2019a, 2020b)<br>Kilosort 1.0 and 2.0<br>Phy and Phy2<br>Fiji (Win 64-bit, ImageJ 1.53f51)

For manuscripts utilizing custom algorithms or software that are central to the research but not yet described in published literature, software must be made available to editors and reviewers. We strongly encourage code deposition in a community repository (e.g. GitHub). See the Nature Portfolio guidelines for submitting code & software for further information.

Data

Policy information about availability of data

All manuscripts must include a data availability statement. This statement should provide the following information, where applicable:

- Accession codes, unique identifiers, or web links for publicly available datasets
- A description of any restrictions on data availability
- For clinical datasets or third party data, please ensure that the statement adheres to our policy

Source data for all figures are provided with this paper as a supplementary data file. Other data that support the findings of this study are available from the corresponding authors upon reasonable request.

Field-specific reporting

Please select the one below that is the best fit for your research. If you are not sure, read the appropriate sections before making your selection.

☒ Life sciences ☐ Behavioural & social sciences ☐ Ecological, evolutionary & environmental sciences

For a reference copy of the document with all sections, see nature.com/documents/nr-reporting-summary-flat.pdf

Life sciences study design

All studies must disclose on these points even when the disclosure is negative.

Sample size	No methods were used to predetermine sample sizes, but our samples sizes reflect those in previous publications. Due to the high-yield nature of our population unit recordings, often only a few animals are enough to test statistical difference. Nevertheless, to confirm the robustness of our findings across animals, at least 4-5 mice were used in each unit recording experiment (Adesnik et al., Nature 490, 226; Reinhold et al., Nat Neurosci 18, 1789). The exact number of animals are given in the main text and also in figure legends.
Data exclusions	We used automated spike sorting system (Kilosort 2) for the isolation of single- and multi-unit activities. Isolated clusters that did not display normal spike shapes were excluded as noise. Units that did not exhibit statistically significant responses to any auditory stimuli are reported, but their tuning was not analyzed. These exclusion criteria are standard in the field and were pre-established before experiments.
Replication	Results described in the paper were reproduced in multiple rounds of experimentations. Data were always acquired from multiple mice and multiple litters. No issues were identified in reproducing any of the reported findings, as shown in the scatter plots or the Source Data. The exact number of repetitions are indicated in text, figure legends, and Supplementary Data 1.
Randomization	Optogenetic manipulation and sound presentations with various frequencies were performed in randomly interleaved trials. Mice were randomly allocated to experiment groups for optogenetic activation (ChrimsonR expression) and inactivation (eNpHR3 expression).
Blinding	A critical step in which bias can potentially occur is in spike sorting, in which individual units are included or excluded in single- or multi-units. We performed spike sorting blind to any stimulus-related information. Thus, a unit's properties were not known at the time of spike sorting. Blinding between activation and inactivation mouse groups was not possible since photostimulation for ChrimsonR and eNpHR3 needed different illumination wavelengths. Nonetheless, optogenetic manipulation was performed in interleaved trials, which ensured the appropriate internal control within each animal. Comparisons were always made between trials with and without optogenetic manipulations and not between animal groups.

Reporting for specific materials, systems and methods

We require information from authors about some types of materials, experimental systems and methods used in many studies. Here, indicate whether each material, system or method listed is relevant to your study. If you are not sure if a list item applies to your research, read the appropriate section before selecting a response.

Materials & experimental systems

n/a	Involved in the study	
☒	☐	Antibodies
☒	☐	Eukaryotic cell lines
☒	☐	Palaeontology and archaeology
☐	☒	Animals and other organisms
☒	☐	Human research participants
☒	☐	Clinical data
☒	☐	Dual use research of concern

Methods

n/a	Involved in the study	
☒	☐	ChIP-seq
☒	☐	Flow cytometry
☒	☐	MRI-based neuroimaging

Animals and other organisms

Policy information about studies involving animals; ARRIVE guidelines recommended for reporting animal research

Laboratory animals	Mice were at least 6 weeks old at the time of experiments. Mice were acquired from Jackson Laboratories (Ai9, C57BL/6J, and CBA/J) or MMRRC (Rbp4-Cre KL100 and Tlx3-Cre PL56). Both female and male animals were used. In some experiments, newborn mice (postnatal day 1-3) were injected with viral vectors, and these mice were used for experiments after they reached at least 6 weeks old.
Wild animals	This study did not involve wild animals.
Field-collected samples	This study did not involve collections from the field.
Ethics oversight	All procedures were in accordance with the Institutional Animal Care and Use Committee at the University of North Carolina at Chapel Hill as well as guidelines of the National Institutes of Health.

Note that full information on the approval of the study protocol must also be provided in the manuscript.
